# Factors Associated With Patient's Refusal of Recommended Cancer Surgery: Based on Surveillance, Epidemiology, and End Results

**DOI:** 10.3389/fpubh.2021.785602

**Published:** 2022-01-17

**Authors:** Xianglin Hu, Hui Ye, Wangjun Yan, Yangbai Sun

**Affiliations:** ^1^Department of Musculoskeletal Surgery, Fudan University Shanghai Cancer Center, Shanghai, China; ^2^Department of Oncology, Shanghai Medical College, Fudan University, Shanghai, China; ^3^Simmons Comprehensive Cancer Center, University of Texas Southwestern Medical Center, Dallas, TX, United States

**Keywords:** cancer, surgery, refusal, racial disparities, marital status, insurance type

## Abstract

**Objectives:**

Most non-metastatic cancer patients can harvest a preferable survival after surgical treatment, however, patients sometimes refuse the recommended cancer-directed surgery. It is necessary to uncover the factors associated with patent's decision in taking cancer surgery and explore racial/ethnic disparities in surgery refusal.

**Methods:**

Based on the Surveillance, Epidemiology and End Results (SEER)-18 program, we extracted data of non-metastatic cancer patients who didn't undergo surgery. Ten common solid cancers were selected. Four racial/ethnic categories were included: White, black, Hispanic, and Asian/Pacific Islander (API). Primary outcome was patient's refusal of surgery. Multivariable logistic regression models were used, with reported odds ratio (OR) and 95% confidence interval (CI).

**Results:**

Among 318,318 patients, the incidence of surgery refusal was 3.5%. Advanced age, female patients, earlier cancer stage, uninsured/Medicaid and unmarried patients were significantly associated with higher odds of surgery refusal. Black and API patients were more likely to refuse recommended surgery than white patients in overall cancer (black-white: adjusted OR, 1.18; 95% CI, 1.11–1.26; API-white: adjusted OR, 1.56; 95% CI, 1.41–1.72); those racial/ethnic disparities narrowed down after additionally adjusting for insurance type and marital status. In subgroup analysis, API-white disparities in surgery refusal widely existed in prostate, lung/bronchus, liver, and stomach cancers.

**Conclusions:**

Patient's socioeconomic conditions reflected by insurance type and marital status may play a key role in racial/ethnic disparities in surgery refusal. Oncological surgeons should fully consider the barriers behind patient's refusal of recommended surgery, thus promoting patient-doctor shared decision-making and guiding patients to the most appropriate therapy.

## Introduction

Although patients with non-metastatic cancers have an opportunity to receive surgery and subsequently harvest a preferable survival, there is always no cancer-directed surgery available to them. It remains unknown which and how many reasons for non-cancer-directed surgery exist in various non-metastatic cancers. The patient's refusal of recommended surgery is one of the modifiable reasons that should be addressed. It has been found that black patients are more likely to refuse recommended surgery than white patients in several cancers (1–3). However, it is unclear whether black-white disparities in refusal are largely mediated by patient's socioeconomic status, such as insurance type and marital status. Therefore, this study seeks to explore the factors that associate with the patent's decision in taking recommended cancer surgery. This study also seeks to explore whether that racial/ethnic disparity in refusal could be narrowed down by controlling the factors such as insurance type and marital status.

## Methods

### Study Population

This population-based cohort study was based on the Surveillance, Epidemiology and End Results (SEER)-18 Registries (Nov 2018 Sub). SEER program covers 28% of US population and collects data on different cancers from population-based cancer registries (https://seer.cancer.gov/). SEER^*^Stat software 8.3.8 was used to retrieve data (https://seer.cancer.gov/seerstat/). Ten common solid cancers were selected: Prostate, lung/bronchus, liver/IBD, pancreas, breast, colorectal, oral cavity/pharynx, stomach, kidney/renal pelvis, and uterus. The inclusion criteria were as follows: (1) Adult patients diagnosed from 2007 to 2015 (2) Non-metastatic cancer patients with the American Joint Committee on Cancer (AJCC) stage I-III; (3) Patients who didn't receive cancer surgery; and (4) Patients with completed data on age, sex, marital status, and insurance type. Patients missing racial/ethnic information were excluded. American Indian/Alaska Native patients were also excluded for limited numbers. A flow diagram was shown in Supplementary Figure 1. Ultimately, 318,318 patients were included.

### Variables

Patient age, sex, race/ethnicity, cancer primary site, AJCC stage, marital status and insurance coverage type were retrieved. Patient survival status and cancer specific survival (CSS) were also retrieved. Four racial/ethnic categories were included: white (non-Hispanic), black (non-Hispanic), Hispanic, and Asian/Pacific Islander (API) (4). Reasons for no cancer-directed surgery were retrieved (5), including: (1) recommended but unperformed surgery, patient refused; (2) recommended but unperformed surgery, unknown reason; (3) patient died before recommended surgery; and (4) not recommended for cancer surgery. Patients with recommended but unperformed surgery due to refusal were compared with patients with recommended but unperformed surgery due to other reason. The primary outcome was patient's refusal.

### Statistical Analysis

Frequencies of different reasons for no-cancer-directed surgery were depicted by cancer type and race/ethnicity. Association of race/ethnicity with surgery refusal was measured using multivariable logistic regression model by stepwise adjustment of insurance and marital status. In multivariable model A, racial association with surgery refusal was assessed by adjusting for age, sex, cancer site, and AJCC stage. In multivariable model B, racial association with surgery refusal was assessed by additionally adjusting for insurance status. In multivariable model C, racial association with surgery refusal was assessed by additionally adjusting for insurance and marital status. In subgroup analysis, racial association with surgery refusal was also assessed in every selected cancer type. Adjusted odds ratios (OR) and 95% confidence intervals (CI) were reported. Association of surgery refusal with cancer specific survival (CSS) was measured using multivariable Cox regression. Adjusted hazard ratio (HR) with 95% confidence interval (CI) was reported. *P* < 0.05 was statistically significant. Statistics and graphics were conducted using SPSS 24.0 and Graph-pad Prism 7.0.

## Results

### Descriptives of the Four Reasons for No Cancer-Direct Surgery

**Figure 1:**
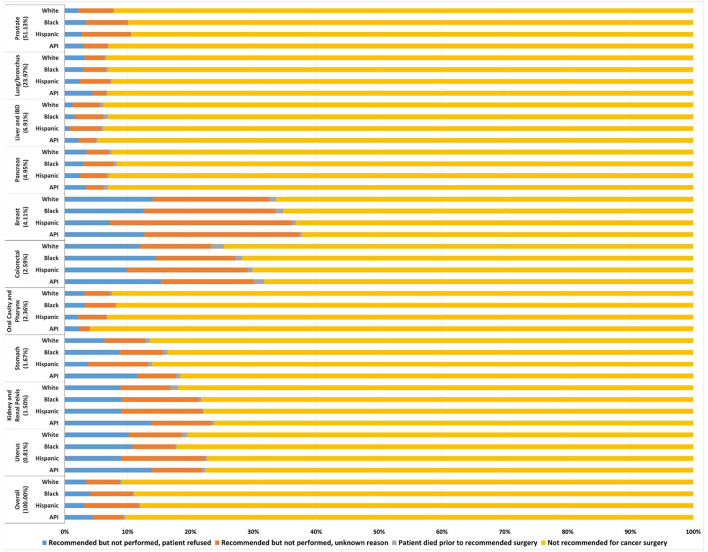
Frequency of the four reasons for no cancer-directed surgery for non-metastatic cancers. Each reason proportion is presented by the four racial and ethnic categories in 10 primary cancers. API, Asian/Pacific Islander; IBD, liver/intrahepatic bile duct.

There were 318,318 stage I-III cancer patients who didn't receive surgery (mean age, 68.1 years; 243,646 [76.5%] male; 217,154 [68.2%] white, 50,370 [15.8%] black, 30,134 [9.5%] Hispanic and 20,660 [6.5%] Asian/Pacific Islander [API]; 85,069 [26.7%] stage I, 154,882 [48.7%] stage II and 78,367 [24.6%] stage III; 162,768 [51.13%] prostate, 76,290 [23.97%] lung/bronchus, 21,982 [6.91%] liver/IBD, 15,762 [4.95%] pancreas, 13,095 [4.11%] breast, 8,251 [2.59%]colorectal, 7,498 [2.36%] oral cavity/pharynx, 5,310 [1.67%] stomach, 4,779 [1.50%] kidney/renal pelvis, and 2,583 [0.81%] uterus). Overall, the incidence of recommended but unperformed surgery was 9.4% (29,932/318,318); it was more prevalent in breast (33.6%), colorectal (25.0%), uterus (19.2%), and kidney/renal pelvis (18.4%) cancers. Overall, the incidence of patient's refusal of recommended surgery was 3.5% (11,221/318,318); it was more prevalent in breast (12.6%), colorectal (12.2%), uterus (10.3%), and kidney/renal pelvis (9.1%) cancers (Figure 1).

### Factors Associated With Patient's Refusal

Among the 29,932 patients with recommended but unperformed surgery, 11,221 (37.49%) patients were due to refusal and 18,711 (62.51%) patients were due to other unspecific reason. The demographic and clinical characteristics of the 29,932 patients were presented in Table 1.

**Table 1:** Demographic and clinical characteristics of patients with recommended but unperformed cancer surgery.

	**Overall (*n* = 29,932)**	**Prostate (*n* = 13,647)**	**Lung/bronchus (*n* = 4,872)**	**Liver/IBD (*n* = 1,234)**	**Pancreas (*n* = 1,092)**	**Breast (*n* = 4,403)**	**Colorectal (*n* = 2,066)**	**Oral cavity and pharynx (*n* = 511)**	**Stomach (*n* = 734)**	**Kidney and renal pelvis (*n* = 877)**	**Uterus (*n* = 496)**
**Age (years)**											
<60	7,660 (25.6)	3,007 (22.0)	689 (14.1)	481 (39.0)	195 (17.9)	2,053 (46.6)	515 (24.9)	188 (36.8)	144 (19.6)	194 (22.1)	194 (39.1)
60-69	9,644 (32.2)	5,909 (43.3)	1,159 (23.8)	436 (35.3)	237 (21.7)	848 (19.3)	463 (22.4)	155 (30.3)	152 (20.7)	169 (19.3)	116 (23.4)
70-79	7,488 (25.0)	3,860 (28.3)	1,600 (32.8)	186 (15.1)	326 (29.9)	564 (12.8)	385 (18.6)	89 (17.4)	193 (26.3)	218 (24.9)	67 (13.5)
≥80	5,140 (17.2)	871 (6.4)	1,424 (29.2)	131 (10.6)	334 (30.6)	938 (21.3)	703 (34.0)	79 (15.5)	245 (33.4)	296 (33.8)	119 (24.0)
**Sex**											
Male	20,234 (67.6)	13,647 (100.0)	2,531 (51.9)	978 (79.3)	511 (46.8)	28 (0.6)	1,155 (55.9)	387 (75.7)	467 (63.6)	530 (60.4)	0
Female	9,698 (32.4)	0	2,341 (48.1)	256 (20.7)	581 (53.2)	4,375 (99.4)	911 (44.1)	124 (24.3)	267 (36.4)	347 (39.6)	496 (100.0)
**Race/ethnicity**											
White	19,019 (63.5)	8,618 (63.1)	3,674 (75.4)	599 (48.5)	750 (68.7)	2,506 (56.9)	1274 (61.7)	376 (73.6)	392 (53.4)	541 (61.7)	289 (58.3)
Black	5,435 (18.2)	2,975 (21.8)	634 (13.0)	188 (15.2)	153 (14.0)	777 (17.6)	298 (14.4)	61 (11.9)	115 (15.7)	150 (17.1)	84 (16.9)
Hispanic	3,550 (11.9)	1,501 (11.0)	291 (6.0)	280 (22.7)	111 (10.2)	690 (15.7)	305 (14.8)	35 (6.8)	120 (16.3)	135 (15.4)	82 (16.5)
API	1,928 (6.4)	553 (4.1)	273 (5.6)	167 (13.5)	78 (7.1)	430 (9.8)	189 (9.1)	39 (7.6)	107 (14.6)	51 (5.8)	41 (8.3)
**AJCC stage**											
I	9,364 (31.3)	2,944 (21.6)	1,900 (39.0)	673 (54.5)	429 (39.3)	1,008 (22.9)	804 (38.9)	101 (19.8)	407 (55.4)	702 (80.0)	396 (79.8)
II	15,379 (51.4)	10,476 (76.8)	657 (13.5)	305 (24.7)	495 (45.3)	2,326 (52.8)	645 (31.2)	150 (29.4)	180 (24.5)	104 (11.9)	41 (8.3)
III	5,189 (17.3)	227 (1.7)	2,315 (47.5)	256 (20.7)	168 (15.4)	1,069 (24.3)	617 (29.9)	260 (50.9)	147 (20.0)	71 (8.1)	59 (11.9)
**Insurance status**											
Insured	25,599 (85.5)	12,697 (93.0)	4,071 (83.6)	933 (75.6)	928 (85.0)	3,304 (75.0)	1,627 (78.8)	412 (80.6)	579 (78.9)	686 (78.2)	362 (73.0)
Uninsured/Medicaid	4,333 (14.5)	950 (7.0)	801 (16.4)	301 (24.4)	164 (15.0)	1,099 (25.0)	439 (21.2)	99 (19.4)	155 (21.1)	191 (21.8)	134 (27.0)
**Marital status**											
Married	15,871 (53.0)	8,882 (65.1)	2,041 (41.9)	543 (44.0)	538 (49.3)	1,849 (42.0)	932 (45.1)	232 (45.4)	361 (49.2)	335 (38.2)	158 (31.9)
Unmarried	14,061 (47.0)	4,765 (34.9)	2,831 (58.1)	691 (56.0)	554 (50.7)	2,554 (58.0)	1,134 (54.9)	279 (54.6)	373 (50.8)	542 (61.8)	338 (68.1)

*API, Asian/Pacific Islander; IBD, liver/intrahepatic bile duct; AJCC, American Joint Committee on Cancer.*

*Data are presented by number (%)*.

As shown in Table 2, in patients with recommended but unperformed surgery, black patients were more likely to refuse recommended surgery than white patients (adjusted OR, 1.18; 95% CI, 1.11–1.26). API patients were more likely to refuse recommended surgery than white patients (adjusted OR, 1.56; 95% CI, 1.41–1.72). Those black-white and API-white disparities in surgery refusal narrowed after additionally adjusting for insurance type and marital status. Patients who refused recommended surgery were more likely to have worse CSS than those who unperformed recommended surgery due to other unspecific reason (adjusted HR, 1.15; 95% CI, 1.10–1.21). In addition, surgery refusal probability significantly increased along with age increase. Female patients were more likely to refuse surgery than male patients (adjusted OR, 1.21; 95% CI, 1.12–1.31). Patients with high cancer stage were less likely to refuse surgery than those with early cancer stage (adjusted OR, 0.55; 95% CI, 0.51–0.59). Uninsured/Medicaid patients were more likely to refuse surgery than insured patients (adjusted OR, 1.41; 95% CI, 1.31–1.52). Unmarried patients were more likely to refuse surgery than married patients (adjusted OR, 1.23; 95% CI, 1.17–1.30).

**Table 2:** Factors associated with patient's refusal of recommended cancer surgery (the outcome is patient's refusal).

**Characteristics**	**Recommended but not performed cancer surgery**		**Crude model**		**Adjusted model**					
	**Other reason (%)**	**Patient refused** **(%)**			**Multivariable model A**		**Multivariable model B (multivariable model A + insurance status)**		**Multivariable model C (multivariable model A + insurance + marital status)**	
	***n* = 18,711**	***n* = 1,1221**	**OR (95% CI)**	***P***	**OR (95% CI)**	***P***	**OR (95% CI)**	***P***	**OR (95% CI)**	***P***
**Age (years)**										
<60	5,447 (71.1)	2,213 (28.9)	1.00		1.00		1.00		1.00	
60–69	6,587 (68.3)	3,057 (31.7)	1.14 (1.07–1.22)	<0.001	1.21 (1.13–1.29)	<0.001	1.24 (1.16–1.33)	<0.001	1.23 (1.15–1.32)	<0.001
70–79	4,666 (62.3)	2,822 (37.7)	1.49 (1.39–1.59)	<0.001	1.45 (1.35–1.56)	<0.001	1.52 (1.41–1.63)	<0.001	1.51 (1.41–1.63)	<0.001
≥80	2,011 (39.1)	3,129 (60.9)	3.83 (3.55–4.13)	<0.001	3.04 (2.81–3.29)	<0.001	3.22 (2.97–3.49)	<0.001	3.13 (2.88–3.39)	<0.001
**Sex**										
Male	13,503 (66.7)	6,731 (33.3)	1.00		1.00		1.00		1.00	
Female	5,208 (53.7)	4,490 (46.3)	1.73 (1.65–1.82)	<0.001	1.27 (1.17–1.38)	<0.001	1.26 (1.16–1.36)	<0.001	1.21 (1.12–1.31)	<0.001
**Race/ethnicity**										
White	11,680 (61.4)	7,339 (38.6)	1.00		1.00		1.00		1.00	
Black	3,409 (62.7)	2,026 (37.3)	0.95 (0.89–1.01)	0.080	1.18 (1.11–1.26)	<0.001	1.14 (1.07–1.22)	<0.001	1.10 (1.03–1.18)	0.005
Hispanic	2,622 (73.9)	928 (26.1)	0.56 (0.52–0.61)	<0.001	0.65 (0.59–0.70)	<0.001	0.60 (0.55–0.66)	<0.001	0.60 (0.55–0.66)	<0.001
API	1,000 (51.9)	928 (48.1)	1.48 (1.34–1.62)	<0.001	1.56 (1.41–1.72)	<0.001	1.49 (1.35–1.64)	<0.001	1.54 (1.39–1.70)	<0.001
**Cancer site**										
Prostate	9,628 (70.6)	4,019 (29.4)	1.00		1.00		1.00		1.00	
Lung/bronchus	2,417 (49.6)	2,455 (50.4)	2.43 (2.28–2.60)	<0.001	2.00 (1.82–2.19)	<0.001	1.91 (1.74–2.09)	<0.001	1.86 (1.70–2.04)	<0.001
Liver/IBD	943 (76.4)	291 (23.6)	0.74 (0.65–0.85)	<0.001	0.70 (0.61–0.81)	<0.001	0.66 (0.57–0.77)	<0.001	0.64 (0.55–0.74)	<0.001
Pancreas	579 (53.0)	513 (47.0)	2.12 (1.87–2.40)	<0.001	1.51 (1.32–1.74)	<0.001	1.46 (1.27–1.68)	<0.001	1.45 (1.26–1.67)	<0.001
Breast	2,753 (62.5)	1,650 (37.5)	1.44 (1.34–1.54)	<0.001	1.15 (1.02–1.28)	<0.001	1.09 (0.97–1.22)	0.134	1.08 (0.97–1.21)	0.161
Colorectal	1,057 (51.2)	1,009 (48.8)	2.29 (2.08–2.51)	<0.001	1.80 (1.61–2.00)	<0.001	1.70 (1.53–1.90)	<0.001	1.68 (1.50–1.87)	<0.001
Oral cavity and pharynx	290 (56.8)	221 (43.2)	1.83 (1.53–2.18)	<0.001	2.00 (1.66–2.42)	<0.001	1.91 (1.58–2.31)	<0.001	1.86 (1.53–2.25)	<0.001
Stomach	372 (50.7)	362 (49.3)	2.33 (2.01–2.71)	<0.001	1.68 (1.42–1.97)	<0.001	1.59 (1.35–1.88)	<0.001	1.57 (1.34–1.85)	<0.001
Kidney and renal pelvis	443 (50.5)	434 (49.5)	2.35 (2.05–2.69)	<0.001	1.57 (1.35–1.83)	<0.001	1.48 (1.27–1.73)	<0.001	1.44 (1.24–1.68)	<0.001
Uterus	229 (46.2)	267 (53.8)	2.79 (2.33–3.35)	<0.001	1.92 (1.56–2.36)	<0.001	1.81 (1.47–2.22)	<0.001	1.77 (1.44–2.18)	<0.001
**AJCC stage**										
I	5,124 (54.7)	4,240 (45.3)	1.00		1.00		1.00		1.00	
II	10,265 (66.7)	5,114 (33.3)	0.60 (0.57–0.64)	<0.001	0.81 (0.76–0.86)	<0.001	0.81 (0.76–0.86)	<0.001	0.81 (0.76–0.86)	<0.001
III	3,322 (64.0)	1,867 (36.0)	0.68 (0.63–0.73)	<0.001	0.55 (0.51–0.60)	<0.001	0.55 (0.51–0.59)	<0.001	0.55 (0.51–0.59)	<0.001
Insurance status										
Insured	16269 (63.6)	9330 (36.4)	1.00		–	–	1.00		1.00	
Uninsured/medicaid	2442 (56.4)	1891 (43.6)	1.35 (1.27–1.44)	<0.001	–	–	1.47 (1.37–1.58)	<0.001	1.41 (1.31–1.52)	<0.001
**Marital status**										
Married	1,0665 (67.2)	5,206 (32.8)	1.00		–	–	–	–	1.00	
Unmarried	8,046 (57.2)	6,015 (42.8)	1.53 (1.46–1.61)	<0.001	–	–	–	–	1.23 (1.17–1.30)	<0.001

*API, Asian/Pacific Islander; IBD, liver/intrahepatic bile duct; AJCC, American Joint Committee on Cancer.*

*Multivariable model A adjusted for age, sex, cancer site, and AJCC stage.*

*Multivariable model B additionally adjusted for insurance status.*

*Multivariable model C additionally adjusted for insurance and marital status*.

### Racial/Ethnic Disparity in Refusal in Every Selected Cancer Type

As shown in Table 3, in subgroup analysis, black patients were more likely to refuse surgery than white patients in prostate (adjusted OR, 1.15; 95% CI, 1.05–1.26) and colorectal cancers (adjusted OR, 1.39; 95% CI, 1.06–1.83); API patients were more likely to refuse surgery than white patients in prostate (adjusted OR, 1.97; 95% CI, 1.65–2.35), lung/bronchus (adjusted OR, 1.93; 95% CI, 1.46–2.54), liver/IBD (adjusted OR, 2.77; 95% CI, 1.90–4.04), and stomach (adjusted OR, 1.97; 95% CI, 1.23–3.15) cancers. Those black-white and API-white disparities in surgery refusal narrowed after additionally adjusting for insurance type and marital status in those specific cancer types.

**Table 3:** Adjusted OR (95% CI) for patient's refusal of surgery in every selected cancer type.

	**Prostate (*n* = 13,647)**	**Lung/bronchus (*n* = 4,872)**	**Liver/IBD (*n* = 1,234)**	**Pancreas (*n* = 1,092)**	**Breast (*n* = 4,403)**	**Colorectal (*n* = 2,066)**	**Oral cavity and pharynx (*n* = 511)**	**Stomach (*n* = 734)**	**Kidney and renal pelvis (*n* = 877)**	**Uterus (*n* = 496)**
Race/ethnicity	Multivariable model 1 (adjusted for age, sex, and AJCC stage)									
White	1.00	1.00	1.00	1.00	1.00	1.00	1.00	1.00	1.00	1.00
Black	**1.15 (1.05–1.26)**	0.96 (0.80–1.15)	1.39 (0.94–2.07)	0.84 (0.56–1.25)	1.16 (0.97–1.40)	**1.39 (1.06–1.83)**	1.01 (0.56–1.80)	1.45 (0.93–2.27)	0.84 (0.57–1.24)	1.46 (0.87–2.43)
Hispanic	**0.86 (0.76–0.97)**	**0.46 (0.35–0.60)**	**0.49 (0.33–0.75)**	0.64 (0.41–1.02)	**0.49 (0.39–0.61)**	**0.67 (0.51–0.89)**	0.57 (0.26–1.24)	**0.45 (0.28–0.72)**	0.75 (0.50–1.13)	0.68 (0.40–1.16)
API	**1.97 (1.65–2.35)**	**1.93 (1.46–2.54)**	**2.77 (1.90–4.04)**	1.15 (0.69–1.91)	1.17 (0.93–1.47)	1.24 (0.89–1.72)	1.88 (0.93–3.79)	**1.97 (1.23–3.15)**	1.33 (0.72–2.46)	1.62 (0.80–3.26)
	Multivariable model 2 (additionally adjusted for insurance type and marital status based on multivariable model 1)									
White	1.00	1.00	1.00	1.00	1.00	1.00	1.00	1.00	1.00	1.00
Black	**1.14 (1.04–1.25)**	0.84 (0.70–1.02)	1.18 (0.78–1.77)	0.74 (0.50–1.11)	1.03 (0.86–1.25)	1.32 (1.00–1.74)	0.94 (0.52–1.70)	1.24 (0.78–1.98)	0.81 (0.54–1.19)	1.42 (0.84–2.39)
Hispanic	**0.82 (0.73–0.94)**	**0.40 (0.30–0.52)**	**0.45 (0.30–0.69)**	**0.57 (0.36–0.92)**	**0.45 (0.35–0.56)**	0.66 (0.49–0.87)	0.47 (0.21–1.05)	**0.39 (0.24–0.64)**	0.68 (0.45–1.04)	0.69 (0.40–1.18)
API	**1.92 (1.61–2.29)**	**1.87 (1.41–2.48)**	**2.52 (1.70–3.74)**	1.05 (0.62–1.77)	1.16 (0.92–1.47)	1.26 (0.90–1.75)	2.01 (0.97–4.15)	**1.85 (1.13–3.00)**	1.23 (0.66–2.28)	1.63 (0.81–3.30)

*API, Asian/Pacific Islander; IBD, liver/intrahepatic bile duct; AJCC, American Joint Committee on Cancer.*

*Data are presented by adjusted OR (95% CI).*

*Bold values indicate statistical significance*.

## Discussion

Nowadays, racial and ethnic inequality in healthcare has garnered widespread attention in the United States. Sometimes it's even described as a serious and shameful public health crisis. Different from other disparities in healthcare, racial and ethnic disparity is rooted in history and modern times, involving multiple elements, such as medical systems, infrastructures, administrative processes, healthcare providers, and individualized patients (6). Occasionally, the term “structural racism” is used to describe racial and ethnic disparities in cancer surgical treatment (7, 8). It remains a key question whether racial and ethnic disparities in cancer surgery utilization are due to structural racism or just socioeconomic status.

Unlike previous studies (1–3), our study compared patients with recommended but unperformed surgery due to refusal to those with recommended but unperformed surgery due to other reason. We found that racial and ethnic disparity in surgery refusal, particularly between black and white patients, was largely mediated by socioeconomic status, like insurance type and marital status. It is well established that cancer patients who refuse surgery have worse survival than those undergoing surgery (9–11). Our study revealed that even when surgery is recommended but unperformed, the patient with refusal would suffer a worse CSS. This might be because patients refusing recommended surgery might also be negative to other alternative recommended treatments.

For prostate cancer patients, Islam et al. (12) found that ~3.9% patients refuse the suggested surgery; black, single, Medicaid/Medicare-covered, or early-stage prostate cancer patients are more likely to refuse the surgery. Those results are consistent with our present findings in prostate cancer. In our study, about 2.47% (4,019/162,768) prostate cancer patients refused recommended surgery. In addition, we identified that API patient was also a population with high odds of refusing recommended surgery. A latest study by Dee et al. (13) found that black and Asian patients are more likely to refuse locoregional treatment (radiotherapy and surgery) than white patients in prostate cancer; locoregional treatment refusal rate for prostate adenocarcinoma has increased over time. For Lung/bronchus cancer patients, Mehta et al. (14) found that blacks and “other” races are more likely to refuse recommended surgery than whites; refusal of surgery is influenced by county variations. In addition, refusal of surgery is influenced by low educational status in lung cancer (15). In recent years, stereotactic body radiotherapy (SBRT) is considered as an alternative proposal for early stage non-small cell lung cancer patients who refuse surgery (16). Nonetheless, a systematic review suggested that SBRT has an inferior effect on survival and long-term distant control compared with surgery for early stage non-small cell lung cancer (17). To date, only a few study explored refusal of surgery in liver/IBD cancer (18). Our study indicated that API-white disparity in surgery refusal significantly existed in liver/IBD cancer. Tohme et al. (2) and Coffman et al. (19) explored the factors associated with surgery refusal in early-stage and non-metastatic pancreatic cancer, respectively, based on the National Cancer Database (NCDB); both of them identified that treatment at a non-academic/research medical center leads to higher odds of refusing pancreatic cancer-directed surgery. Those results suggested that hospital level is a key factor influencing patient's trust and decision in taking surgery. Much attention has been paid to surgery refusal in colorectal cancer patients (1, 9–11, 20). Surgery refusal rate for non-metastatic colorectal cancer is increasing over time, which increases colorectal cancer-specific mortality (adjusted HR: 5.10, 95% CI: 4.62-5.62) (20). Our study indicated that there was a significant black-white disparity in surgery refusal in non-metastatic colorectal cancer, which was largely mediated by patient's socioeconomic status. Surgery refusal has been also well investigated in breast cancer patients (21–23). Compared to refusals of chemotherapy and endocrine therapy, refusal of surgery causes the highest mortality risk for patients with invasive breast cancer (23). To date, other cancer types such as stomach, oral cavity/pharynx, kidney/renal pelvis and uterus cancers, are relatively less reported in surgery refusal, which warrants reinforced studied in the future.

Some limitations should be noticed in this SEER-based population study. First, this study is retrospective, it is intriguing to know whether and which complementary therapy that the patients would choose after refusing the recommended surgery. Secondly, in this pan-cancer analysis, prostate, lung and breast cancer patients account for the majority. The main findings apply to overall cancer population and must be prudently extended to every selected cancer type for potential selection bias. Thirdly, patient's decision in taking recommended surgery involves various influencing factors. It is a patient-doctor mutual participation result. Besides patient's socioeconomic conditions, more factors such as surgeon's conversation skills are warranted to be noticed in the future.

## Conclusion

As early as 2009, the American Society of Clinical Oncology (ASCO) has recognized racial and ethnic disparity as a major obstacle to achieving cancer health equity and listed it in cancer care as a critical issue to conquer. ASCO has issued a series of policies and recommendations to promote cancer health equity, such as ensuring equitable access to high-quality care and research, removing structural barriers, and increasing awareness and action (6). Patient's socioeconomic conditions reflected by insurance type and marital status may play a key role in racial/ethnic disparities in surgery refusal. According to our findings, in the future, oncological surgeons should fully consider the hindrances behind patient's refusal of recommended surgery, thus promoting patient-doctor shared decision-making and guiding patients to the most appropriate therapy.

## Data Availability Statement

Publicly available datasets were analyzed in this study. This data can be found at: https://seer.cancer.gov/.

## Ethics Statement

Ethical review and approval was not required for the study on human participants in accordance with the local legislation and institutional requirements. Written informed consent for participation was not required for this study in accordance with the national legislation and the institutional requirements.

## Author Contributions

WY, HY, and YS contributed to the study conception and revised the manuscript. XH contributed to the data analysis and interpretation and draft the manuscript. All authors contributed to the article and approved the submitted version.

## Conflict of Interest

The authors declare that the research was conducted in the absence of any commercial or financial relationships that could be construed as a potential conflict of interest.

## Publisher's Note

All claims expressed in this article are solely those of the authors and do not necessarily represent those of their affiliated organizations, or those of the publisher, the editors and the reviewers. Any product that may be evaluated in this article, or claim that may be made by its manufacturer, is not guaranteed or endorsed by the publisher.
