# Supplementary figures and images for: Factors Associated With Patient's Refusal of Recommended Cancer Surgery: Based on Surveillance, Epidemiology, and End Results
Source: Front Public Health. 2022 Jan 17;9:785602. doi: 10.3389/fpubh.2021.785602 (PMC8801711; doi:10.3389/fpubh.2021.785602)

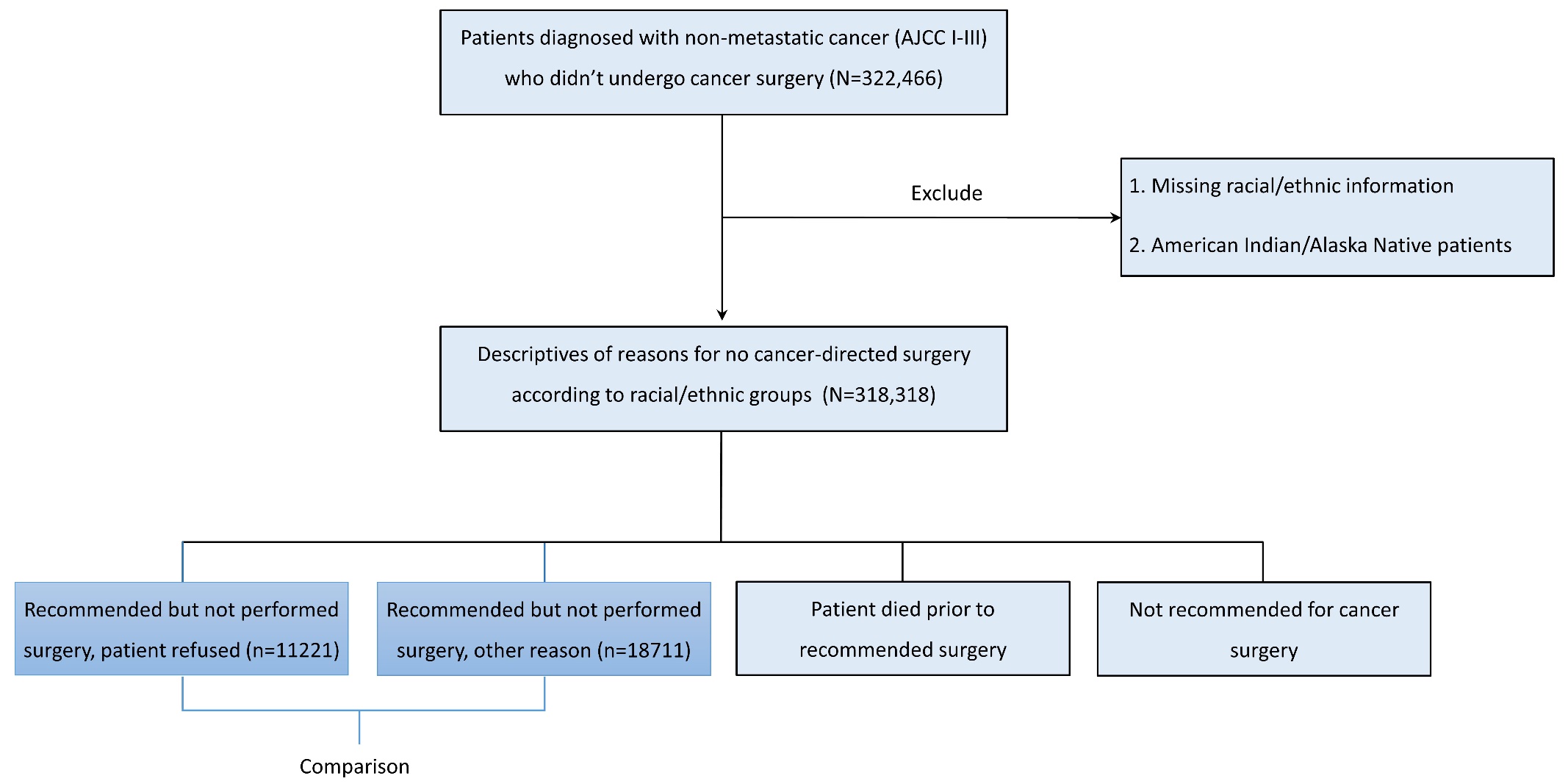


**Supplementary Figure 1. Flow diagram showing patient enrollment.**

Supplement: Supplementary file 1 [file Table_1.DOCX]
